# Use of Doppler ultrasound renal resistive index and neutrophil gelatinase-associated lipocalin in prediction of acute kidney injury in patients with septic shock

**DOI:** 10.1186/cc9528

**Published:** 2011-03-11

**Authors:** CW Ngai, MF Lam, SH Lo, CW Cheung, WM Chan

**Affiliations:** 1 Adult Intensive Care Unit, Queen Mary Hospital, Pokfulam, Hong Kong; 2 Department of Medicine, Queen Mary Hospital, Pokfulam, Hong Kong; 3 Department of Surgery, Queen Mary Hospital, Pokfulam, Hong Kong; 4 Department of Anaesthesiology, The University of Hong Kong, Pokfulam, Hong Kong

## Introduction

Acute kidney injury (AKI) is common in septic shock and there is no good marker to predict it. Neutrophil gelatinase-associated lipocalin (NGAL) is a novel renal biomarker showing promising results in prediction of AKI in patients across different clinical settings. Another potential marker is the resistive index (RI) of renal interlobar artery (calculated as (peak systolic velocity - end diastolic velocity)/peak systolic velocity), which has been shown to be useful in identifying those who will develop AKI in patients with septic shock. The aim of this study is to evaluate the usefulness of RI and NGAL in the early detection of AKI.

## Methods

A prospective, observational study in a 20-bed medical/surgical ICU of a university teaching hospital. All patients with septic shock were recruited, excluding those with chronic renal failure (serum creatinine >120 μmol/l). Within the first 24 hours after the introduction of vasopressor, urine and serum were collected for NGAL measurement and RI was determined by two independent operators. The occurrence of AKI was measured at day 3, according to RIFLE criteria. RI and NGAL were compared between patients with (RIFLE-F) and without (RIFLE-0/R/I) AKI.

## Results

During the period from August to November 2010, 20 patients (age 58 ± 16) with septic shock were recruited. Eleven patients were classified as having AKI. No significant difference in baseline characteristics such as APACHE II score and baseline creatinine was shown at enrollment. RI, serum-NGAL and urine-NGAL were all higher in patients with AKI (RI: 0.749 ± 0.0697 (mean ± SD) vs. 0.585 ± 0.0983, *P *< 0.001; serum-NGAL: 2,182 ± 838 ng/ml (mean ± SD) vs. 1,075 ± 1,006, *P *= 0.015; urine-NGAL: 2,009 ± 3,370 vs. 993 ± 1,789 (median ± IQR), *P *= 0.025). Area under the ROC curve for RI and serum-NGAL was 0.909 (± 0.088, *P *= 0.002) and 0.808 (± 0.113, *P *= 0.02), respectively. For RI, using 0.65 as the cut-off, sensitivity and specificity was 1 and 0.89, respectively. For serum-NGAL, using a cut-off of 1,200 ng/ml, it had a sensitivity of 1 and specificity of 0.67. Inter-observer difference of RI was low (0.0015 ± 0.0074 (mean ± SD)).

## Conclusions

Doppler ultrasound renal RI is non-invasive, rapidly available and easily reproducible, and is at least as good as NGAL as a predictor of AKI in patients with septic shock.
